# Risk Factors for Trimalleolar Ankle Fracture: A Single-Centre Retrospective Analysis

**DOI:** 10.7759/cureus.98158

**Published:** 2025-11-30

**Authors:** Oliver M Scarborough, Cameron Kennedy, Owen Morris, Amiera Mokhsin, Seied Amir Bafi Ghadimi, Chi Hoi Lee, Omar Toma

**Affiliations:** 1 Trauma and Orthopaedics, Manchester University NHS Foundation Trust, Manchester, GBR; 2 Orthopaedics, Aintree University Hospital, Liverpool, GBR; 3 Trauma and Orthopaedics, Wrexham Maelor Hospital, Wrexham, GBR; 4 Critical Care, Manchester University NHS Foundation Trust, Manchester, GBR

**Keywords:** ankle fracture, ankle fracture surgery, bone mineral density, obesity, post-menopause, trimalleolar ankle fracture

## Abstract

Background

Obesity presents a growing public health crisis within the UK. Recent literature has identified a direct correlation between obesity and the incidence of ankle fractures in both men and women. In addition to this, the reported incidence of severe ankle fractures and those requiring operative fixation in the obese population is significantly higher than that in the non-obese population. This study aims to investigate associations between obesity and tri-malleolar ankle fracture, specifically, while reviewing any potential differences in gender. We will also examine the fracture rates in post-menopausal women to determine if menopause is an independent risk factor for trimalleolar ankle fracture.

Method

Search terms were used to identify all operatively managed ankle fractures listed on the virtual trauma board at a major trauma centre in Manchester, UK. The records of each of these patients were then reviewed on an individual basis to identify all trimalleolar ankle fractures (161 patients). Patient demographics and injury-specific data were collected and input into a data sheet using Microsoft Excel (Microsoft® Corp., Redmond, WA).

Results

Obesity and severe obesity were common and seen in 60 (37.3%) and 11 (6.8%) patients, respectively. Our cohort was found to have a significantly higher mean BMI (30.6 SEM = 0.59) than the national average (27.6) (p < 0.001). Women had a higher average BMI at 31.8, whereas the average BMI for men was 28.4. There were 49 (46.7%) postmenopausal women (aged ≥ 55 years old) in our sample size of 161 patients. This was significantly more than there were in the general population for the same sample size (p < 0.001).

Conclusion

Our paper demonstrates an association between obesity and trimalleolar ankle fracture. Furthermore, women were more prone to trimalleolar ankle fractures than men. However, we are unable to say if female gender is an independent risk factor since women in our study had a higher average BMI than the men. The findings around bone mineral density (BMD) are conflicting, and although there were many postmenopausal women in our cohort, recent studies suggest that menopause does not play a role in increasing the risk of ankle fracture. Further research should focus on the role of menopause as a risk factor in trimalleolar ankle fracture.

## Introduction

Obesity, defined by a body mass index (BMI) over 30 kg/m^2^ [1], presents a growing public health crisis within the UK. Over a quarter of the UK population is now classified as obese (26.5%), with nearly two-thirds either overweight or obese (64.5%) [1,2]. Despite recent advancements in bariatric surgeries and the novel introduction of weight reduction medications, current projections indicate this incidence is going to increase [3,4]. In orthopaedics, obese patients have a higher risk of developing osteoarthritis, sustaining fractures and have poorer post-operative outcomes due to increased risk of infection, blood clots and metalwork failure [5].

Ankle fractures are the most common type of lower limb fracture and one of the most common fractures worldwide. They follow a bimodal distribution, affecting young males and elderly females [6]. Recent literature has identified a direct correlation between obesity and the incidence of ankle fractures in both men and women [7]. In addition to this, the reported incidence of ankle fractures requiring operative fixation in the obese population is significantly higher than that in the non-obese population [8,9]. Furthermore, a recent retrospective study by Wong et al. suggests that obesity is associated with increasing severity of ankle fracture patterns [10]. The increased weight an obese patient carries causes additional strain and load to their skeleton and may also cause skeletal misalignment in the lower limbs, predisposing them to falls [5,10]. Although not fully understood, it is thought that for these reasons, obesity may contribute to a higher incidence of lower limb fractures [5,11,12]. Our paper will examine trimalleolar fractures specifically, because of their higher surgical burden and poorer outcomes [10].

There are important gender differences in the incidence of ankle fractures. In younger adults, high-energy injuries requiring surgical intervention disproportionately affect men [13]. Whereas in patients over the age of 50, women typically sustain more severe ankle injuries due to higher BMI and post-menopausal osteoporosis [13-15]. Notably, the reported incidence of ankle fractures in obese post-menopausal women has a three-fold increase compared to non-obese post-menopausal women [15]. This suggests that obesity is an independent risk factor for this demographic.

This study has two primary objectives. The first is to investigate associations between obesity and tri-malleolar ankle fracture occurrence, while reviewing any potential differences in gender. With regard to this objective, the authors hypothesise that obesity and female gender will be associated with an increased incidence of trimalleolar ankle fractures. Secondly, we will compare the fracture rates between pre- and post-menopausal women to determine if menopause is an independent risk factor for trimalleolar ankle fracture. Here, the authors hypothesise that postmenopausal status will be associated with higher rates of trimalleolar ankle fracture.

## Materials and methods

Our major trauma centre (MTC) in Manchester, UK, has used a virtual trauma board to list all its trauma cases since it was first introduced to the trust on 08/09/2022. Every patient listed for surgery under the care of the Orthopaedic Team between the aforementioned date and the time of data collection (20/05/2025) was filtered into a research folder. The search term ‘ankle fracture’ was then applied to identify all patients with operatively managed ankle fractures within this time frame. 

The records of each of these patients were reviewed on an individual basis, with imaging and operation notes screened in order to identify all trimalleolar ankle fractures. All patients had a CT scan of the ankle reported by a radiologist, which confirmed the diagnosis. Patients were eligible for inclusion in the study if they were aged 16 years or older and had an operatively managed trimalleolar ankle fracture during the study window. Single malleolar, bimalleolar and pilon fractures were excluded. Children aged less than 16 years were also excluded. 

Injury-specific data collected included high or low energy mechanism, open vs closed injury, date of injury (DOI), date of definitive fixation, the time between injury and fixation and total number of surgeries. Patient demographics included hospital number, date of birth, age, gender, BMI and weight classification. All data was anonymised and included no patient-identifiable information. The data was collected for audit and service evaluation purposes only and was not shared beyond the authors of this paper. Due to the retrospective nature of the study and careful information governance, ethical approval was not required.

The National Institute for Health and Care Excellence (NICE) guidelines on obesity were used to classify the BMI of the patients included in our study [16]. When classifying the energy mechanism as either high or low, any fall from a standing height was classified as low energy, and any injury sustained through a road traffic accident was classified as high energy. For falls from a height, the NICE guidelines for assessment and early management in head injury were used as a guide for classifying the energy of the mechanism [17]. As per their guidance, all falls from a height of more than one metre, or five stairs, were considered to be high-energy. 

Statistical analysis

All data analysis was performed in RStudio version 4.5.1 (Posit, Boston, MA). Data that followed a normal distribution were summarised with mean and standard deviation; categorical data were summarised using counts and percentages. One-sample t-tests were used to compare the continuous variables to the national averages. One proportion z-tests were used to compare proportions in the data to the national proportions. Proportions were compared between groups in the dataset using the two-proportion test. p-values were considered significant if they were less than 0.05. Where there was missing BMI data, the distribution was assessed graphically using qq plots and histograms. Where data was not available, no imputation was used.

## Results

**Figure 1:**
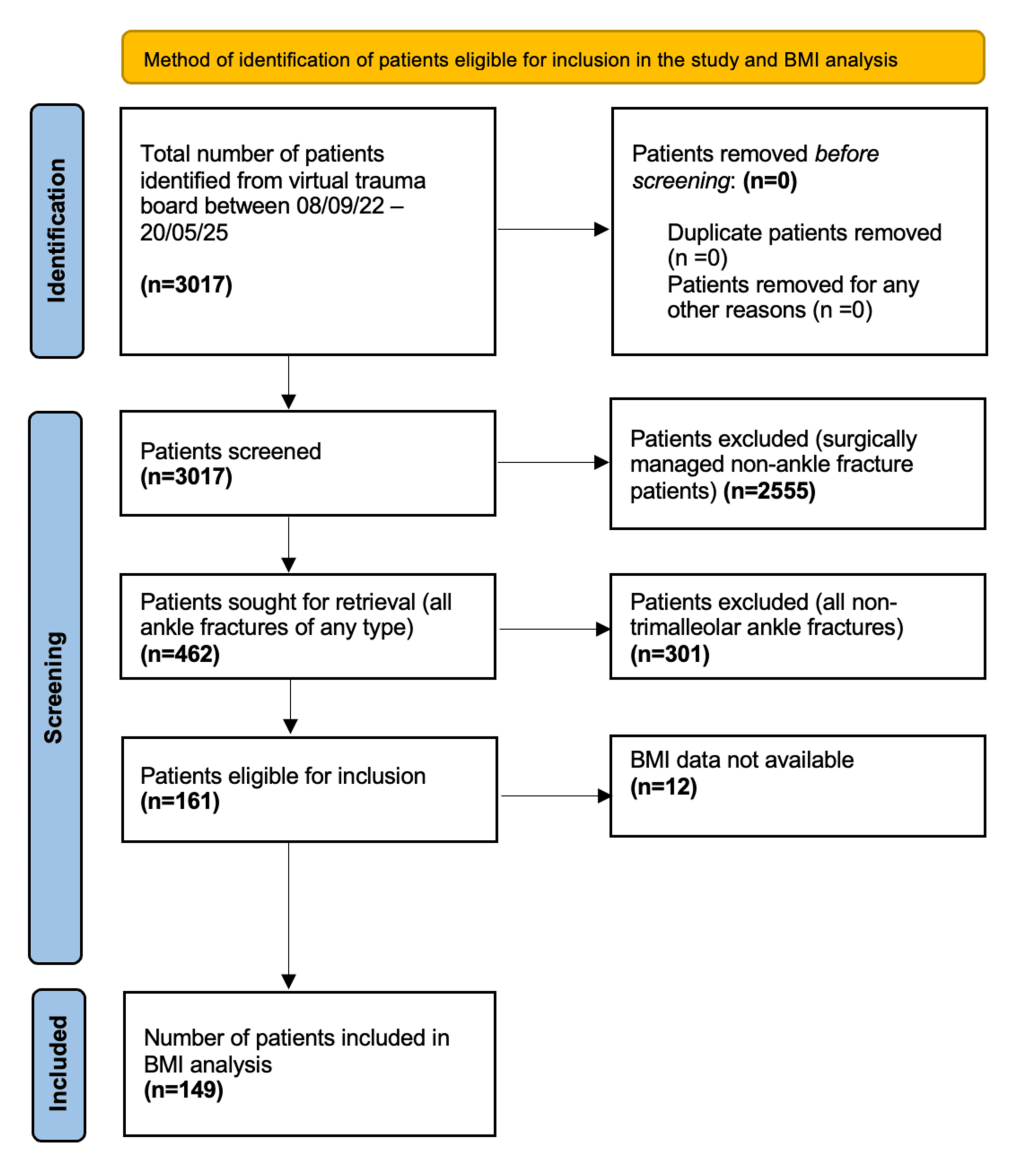
Flow diagram demonstrating the selection process for study inclusion and BMI analysis

The total number of patients listed on the virtual trauma board up until the time of data extraction was 3017. Following application of search terms, 462 patients were identified who had suffered an ankle fracture of any type. The records of each of these patients were reviewed on an individual basis, with imaging and operation notes screened, and the inclusion/exclusion criteria applied. This identified a total of 161 trimalleolar ankle fractures for inclusion in the study. There was no BMI data available for 12 of these patients, because either height or weight (or both) were not recorded at the time of admission. These patients were therefore excluded from the analysis of the BMI results. The participant selection process is demonstrated by the flow diagram in Figure 1.

Table 1 shows the patient demographics of the participants included in our study. Of the 161 patients included, 56 (35%) were male, and 105 (65%) were female. The overall average age was 49.7 years. The average age for men was 46.7 years and 51.3 years for women. The average BMI for men and women combined was 30.6. Women had a higher average BMI at 31.8, whereas the average BMI for men was 28.4. 

**Table 1:** Patient demographics for the male cohort, female cohort and total cohort. p-values were considered significant if they were <0.05. *Denotes significant p-values.

Variable		Male Cohort n = 56	Female Cohort n = 105	Total Cohort n = 161
Age	Mean (SD)	46.7 (16.3)	51.3 (19.8)	49.7 (18.7)
BMI	Mean (SD)	28.4 (6.4)	31.8 (7.5)	30.6 (7.3)
	p-value	0.353	<0.001*	<0.001*
	t-value	0.94	5.51	5.04
	Missing (%)	3 (5.4)	9 (8.6)	12 (7.5)
Weight classification	Underweight (%)	1 (1.8)	0 (0.0)	1 (0.6)
	Healthy weight (%)	15 (26.8)	18 (17.1)	33 (20.5)
	Overweight (%)	18 (32.1)	26 (24.8)	44 (27.3)
	Obese (%)	17 (30.4)	43 (41.0)	60 (37.3)
	Severely obese (%)	2 (3.6)	9 (8.6)	11 (6.8)
	Missing (%)	3 (5.4)	9 (8.6)	12 (7.5)
	Obese and severely obese combined (%)	19 (33.9)	52 (49.5)	71 (44.1)
	Obese and severely obese combined p-value	0.620	<0.001*	<0.001*
	Obese and severely obese combined x-value	0.25	21.10	30.72

Obesity and severe obesity were common and seen in 60 (37.3%) and 11 (6.8%) patients, respectively. Among men, 19 patients (34%) were classified as either obese or severely obese (17 patients (30.4%) and two patients (3.6%) respectively). The numbers were even higher in women, with 52 (49.6%) classified as obese or severely obese (43 patients (41%) and nine patients (8.6%), respectively.

A one-sample t-test was used to compare the BMI in the total cohort to the 2022 national average BMI. This has been repeated for the male cohort and female cohort separately, using the appropriate national average. The total cohort was found to have a significantly higher mean BMI (30.6 SEM = 0.59) than the national average (27.6) (p < 0.001). Mean BMI of the female-only cohort was also significantly higher (31.8, SEM = 0.77) than the 2022 national average for females (27.6) (p < 0.001). There was no significant difference found between the mean BMI of the male-only cohort (28.4, SEM = 0.88) and the 2022 national average for males (0.88) (p = 0.353). A two-sample independent t-test suggests that the mean BMI of the female cohort is significantly higher than that of the male cohort (p = 0.004).

The proportion of obese and severely obese participants in our study was compared to the 2022 national average. The 2022 national average was 32%. A one-proportion z-test determined that there was a significantly higher proportion of obese and severely obese participants in the total cohort (44.1%) (p < 0.001). This was also seen when comparing the proportions of obese and severely obese female patients with the national average for females (49.5% vs 34% respectively) (p < 0.001). However, there was not a significant difference between the proportion of obese and severely obese male participants and the national average for males (33.9% vs 30% respectively) (p = 0.620).

Table 2 shows the injury demographics for the patients included in our study. There were 22 (13.7%) fractures sustained through a high-energy mechanism and 139 (86.3%) through low energy. Of the high-energy patients, 14 (63.6%) were men, and eight (36.4%) were women. A two-proportion test determined that there was a significantly higher proportion of high-energy fractures in the male cohort than the female cohort (14 patients (25.0%) vs eight patients (7.6%) (p = 0.005). In the high-energy mechanism group, 6 (27.3%) patients were classified as obese, and one (4.5%) patient was severely obese. In the low-energy mechanism group, 48.1% were obese or severely obese (56 patients (40.3%) and nine patients (6.5%), respectively).

**Table 2:** Injury demographics for the male cohort, female cohort and total cohort. p-values were considered significant if they were <0.05. *Denotes significant p-values.

Variable		Male Cohort n = 56	Female Cohort n = 105	Total Cohort n = 161
High-energy injury	n (%)	14 (25.0)	8 (7.6)	22 (13.7)
	p-value	0.005*	Not tested	Not tested
	x-value	7.94	Not tested	Not tested
Low-energy mechanism (%)		42 (75.0)	97 (92.4)	139 (86.3)
Open fracture (%)		15 (26.8)	21 (20.0)	36 (22.4)
Closed (%)		41 (73.2)	84 (80.0)	125 (77.6)

Open fractures were present in 36 patients (22.4%), and 125 patients (77.6%) had closed fractures. Of the open fractures, 15 (41.7%) were male, and 21 (58.3%) were female. Of the closed fractures, 41 (32.8%) were male, and 84 (67.2%) were female. The average BMI of open fractures was 32.4, and that of closed fractures was 30.1. 

For the purposes of our study, we inferred menopausal status using a cut-off of 55 years. There were 49 women aged ≥55 years old (46.7%) and 56 women aged ≤55 years old (53.3%). The average BMI for those ≥55 years was 32.7, and the average BMI for those <55 years old was 31.1. Of the 49 female patients aged ≥55 years old, 48 (98%) patients sustained low-energy fractures in comparison to 1 (2%) patient who sustained a high-energy fracture. In the same cohort of women, 16 (32.7%) injuries were open, and 33 (67.3%) were closed. In those <55 years of age, 49 patients (87.5%) suffered the injury through a low-energy mechanism vs seven (12.5%) through a high-energy one. There were five open fractures (8.9%) and 51 closed fractures in these women (91.1%).

The average time between DOI and definitive fixation for all patients combined was 10 days. For open fractures, it was an average of 7.4 days and 14.1 days for closed fractures. The average number of surgeries per patient overall was 1.4.

## Discussion

This study aimed to investigate the association between obesity and trimalleolar ankle fracture, evaluate gender differences and ascertain whether postmenopausal status is a risk factor.

Our findings showed that the average BMI in patients sustaining trimalleolar ankle fracture was 30.6. This is in contrast to the national average for adults, reported as 27.6 by NHS England in 2022 [18]. A large proportion of our cohort was classified as either obese or severely obese (71 patients, 44.1%). A further 44 patients (27.3%) were classified as overweight, leaving just 34 (22.8%) of our cohort classified as either healthy weight (33 patients, 22.1%) or underweight (1 patient, 0.7%). These findings demonstrate an association between obesity and trimalleolar ankle fracture (p < 0.001). Research shows there are several reasons behind this. Firstly, high BMI increases the risk of falls through postural instability and reduced muscle function due to a greater intramuscular fat content [19]. As the probability of falls increases, so does the likelihood of fracture. Secondly, obesity has a direct correlation to ankle fractures due to altered gait changes. These changes are due to abnormal forward progression forces, quadriceps weakening, poor balance and in some cases, acquired flat foot deformity [7]. Finally, the increased weight will result in a greater force associated with the fall, leading to a higher probability of fracture [20]. 

There was a strong preponderance towards women in our cohort, with 105 (65%) of the participants being female and only 56 (35%) being male. The average BMI amongst women was slightly higher at 31.9 compared to 28.4 in men (p < 0.004). Whilst our findings do support current literature that both obesity and female gender appear to be risk factors for ankle fracture [8], whether female gender is an independent risk factor is unclear, as it may be the higher average BMI that carries the greatest risk.

In our study, men were more likely to sustain high-energy injuries resulting in trimalleolar fractures compared to women. This may be due to a higher proportion of men involved in high-energy mechanism incidents, such as road traffic collisions (RTC). Official statistics of UK RTCs in 2023 from the Department for Transport show men were involved in 75% of fatalities and 61% of casualties of all severities [21]. The average BMI of the high-energy mechanism cohort was slightly lower than that of the low-energy one. This may lend weight to the argument that a higher BMI is a risk factor for sustaining trimalleolar fractures during low-energy mechanisms.

In some fractures, it has been shown that increased adipose tissue may provide a cushion to the bony sites, reducing the risk of fracture; however, this is not the case for ankle fractures (15). BMI is a less significant risk factor for high-energy mechanism injuries, as the force from the mechanism alone would be sufficient to result in fracture without the additional force of the patient’s body weight [20]. The high-energy injury cohort was only made up of 22 patients, so it may be the case that with a greater sample size, the proportions become like those of low-energy injuries.

The higher proportion of females overall and especially amongst the low energy mechanism cohort could suggest that bone mineral density (BMD) plays a role. Naturally, BMD falls after menopause [15], and one could hypothesise that the risk of fracture would increase thereafter. However, this is a controversial topic as studies have shown that when BMD is measured, it is not a significant factor [8]. Menopause usually occurs between the ages of 45 and 55, and epidemiological studies demonstrate that <5% of women are pre-menopausal over the age of 55 [22]. We observed in our cohort a high number of women aged over 55 years when compared to the general population. According to the 2021 census by Manchester City Council [23], the number of women aged >55 in the general population at the time was 85,413. This was in relation to a total population of 551,937. Based on these figures, we can assume that in a sample size of 161 people, there will be 24.9 women aged >55 years. However, in our sample of 161 patients, there were 49. This difference was found to be statistically significant (p < 0.001). 

In post-menopausal women, peripheral adipose tissue is the most important source of endogenous oestrogen production, and this increases BMD [15]. For this reason, it had been widely assumed until recently that obesity was protective against fractures [10]. However, many researchers, including Armstrong et al., suggest that the negative effects of obesity and its role in causing falls outweigh any protective effects of obesity on BMD [15]. Given the link between obesity and ankle fracture seen in our study, we also believe this to be true.

Open fractures had a faster average time to definitive fixation compared to closed fractures, with an average of 7.4 days and 14.1 days, respectively. Open fractures require surgical debridement to reduce the risk of infection within 24 hours at most, depending on the nature of contamination and energy of the mechanism [24]. A systematic review by Hulsker et al. found that immediate definitive fixation of open ankle fractures was safe and led to good functional outcomes. They suggest that external fixation should only be considered if there is inadequate soft tissue coverage [25]. However, ankle fractures are often associated with significant swelling, and this may limit the ability to perform immediate fixation, as may the soft tissue coverage [26]. Where the BOAST guidelines for the management of ankle fractures [27] suggest closed fractures should be fixed on day zero or one, a limit of resources in our centre often meant this was not possible, and patients were left waiting for the swelling to subside before their surgery could go ahead.

Strengths and limitations

We acknowledge there are several limitations to our study. The virtual trauma board was used to identify suitable participants, and this gave a limited sample of 161 patients, although this was a sample retrieved over a reasonable time frame of more than two years. The retrospective nature of the study meant that the care of patients included was not altered because of its implementation. However, the findings reflect only our institution’s population, protocols and clinician behaviours, and may not necessarily be generalised to the wider population. No multivariate analysis was performed to adjust for confounders like age or comorbidities, and BMD was not directly measured as part of our study. We recognise that only operatively managed patients were included in the study, and there may have been a cohort of patients who were managed non-operatively that were not included. It is possible that these patients were managed conservatively due to co-morbidities such as poorly controlled diabetes and hypertension, amongst others. These conditions can be associated with obesity, and the inclusion of these patients may have lent further strength to our study. However, the authors were aware of this at the time of study planning, and there was no conceivable way of identifying these patients for inclusion.

## Conclusions

Our paper supports an association between obesity and trimalleolar ankle fracture and suggests the reason behind this is a complex interaction between abnormal biomechanics, increased falls risk and greater force transmitted to the ankle mortise from the fall itself. In our cohort, women are more prone to trimalleolar ankle fractures than men. However, we cannot conclude that female gender is an independent risk factor since women in our study had a higher average BMI than the men. The cause of fracture seems to differ between genders, with more men suffering high-energy injuries. While there was a high proportion of postmenopausal women in our study, the findings around BMD are conflicting and recent studies suggest that menopause does not play a role in increasing the risk of ankle fracture. Our preliminary results provide a good basis for further research and warrant confirmation in larger, multi-centre studies. Further research should focus on the role of menopause as a risk factor in trimalleolar ankle fracture.
